# The Promise of Affective Language for Identifying and Intervening on Psychopathology

**DOI:** 10.1007/s42761-023-00199-w

**Published:** 2023-08-02

**Authors:** Erik C. Nook

**Affiliations:** Department of Psychology, Princeton University, Peretsman Scully Hall, Washington Rd, Princeton, NJ 08540 USA

**Keywords:** Language, Emotion, Emotion Regulation, Mental health, Linguistic distancing

## Abstract

We are in dire need of innovative tools for reducing the global burden of psychopathology. Emerging evidence suggests that analyzing language (i.e., the words people use) can grant insight into an individual's emotional experiences, their ability to regulate their emotions, and even their current experiences of psychopathology. As such, linguistic analyses of people’s everyday word use may be a diagnostic marker of emotional well-being, and manipulating the words people use could foster adaptive emotion regulation and mental health. Given the ubiquity of language in everyday life, such language-based tools for measuring and intervening in emotion and mental health can advance how we identify and treat mental illnesses at a large scale. In this paper, I outline the promise of this approach and identify key problems we must solve if we are to make it a reality. In particular, I summarize evidence connecting language, emotion, and mental health for three key constructs: sentiment (i.e., the valence of one’s language), linguistic distancing (i.e., using language to separate oneself from distressing stimuli), and emotion differentiation (i.e., using words to specifically identify one’s emotions). I also identify open questions in need of attention for each of these constructs and this area of research as a whole. Overall, I believe the future is bright for the application of psycholinguistic approaches to mental health detection and intervention.

Psychopathology accounts for immense human and economic burdens. More than half of Americans will experience a mental disorder, and psychopathology constitutes almost 10% of the global burden of disease (Kessler et al., 2005; Rehm & Shield, 2019). What innovations and insights can affective science offer to battle these staggering statistics? Here, I review research demonstrating that analyzing language (i.e., the words we use) can be a tool for both measuring and manipulating emotion, emotion regulation, and even mental health, focusing on three constructs: sentiment (i.e., the valence of one’s words), linguistic distancing (i.e., using words to separate ourselves from stressors), and emotion differentiation (i.e., using words to specifically identify what we are feeling).

Whether it is crushing sadness, crippling anxiety, destructive anger, or unending emptiness, difficult emotional experiences are central to psychopathology (American Psychiatric Association, 2013; Gross & Jazaieri, 2014). If words provide windows into minds (Jackson et al., 2022), could we tell how someone is feeling based on their language alone? Studies on sentiment have begun to show just that (Wankhade et al., 2022). Linguistic measures of sentiment vary from simply using preset dictionaries to categorize and count words as positive or negative (e.g., using linguistic inquiry and word count (LIWC); Pennebaker et al., 2007), through more complex approaches that examine both the words that are used and their order (e.g., VADER; Hutto & Gilbert, 2014), to machine-learning approaches that analyze a huge number of variables using complex algorithms to classify texts as positive or negative (e.g., support vector machines (SVM); Prabowo & Thelwall, 2009). These measures correlate with self-reported affect (Kahn et al., 2007; Massachi et al., 2020; Nook et al., 2017; Truong et al., 2012), and evidence is growing that they also track symptoms of psychopathology (Burkhardt et al., 2021; Li et al., 2022). For example, the sentiment of a person’s text messages defined using dictionary-based methods correlates with their internalizing symptoms (Stamatis et al., 2022).

However, not all studies have found these relations (Kross et al., 2019; McNeilly et al., 2023). As one example, Kross and colleagues (2019) found that Facebook posts analyzed using a dictionary-based measure of sentiment did not correlate with self-reported affect. These discrepancies highlight key open questions regarding the validity of language-based measures of affect. For instance, do non-replications arise because relations between sentiment and emotion are very small, leading some studies to uncover null relations simply due to chance? Or do they reflect some systematic force, such as the type of sentiment measure used (e.g., LIWC vs. VADER), the type of linguistic data analyzed (e.g., text messages vs. social media posts), or the situation a person is in when language is measured (e.g., a typical workday vs. an initial therapy session)? Each of these factors likely contributes to the mixed results observed in the literature. Some linguistic measures are probably better correlates of affect than others, and context surely moderates the extent to which language tracks emotion and/or mental health (e.g., Aldao, 2013). Indeed, people sometimes use negative words without feeling negative emotions (e.g., friends can enjoy berating a bad movie together), suggesting that sentiment can sometimes have a null or inverse relationship with a person’s affective experience. Nonetheless, these glimmers of initial correlations between sentiment, affect, and psychological symptoms suggest that there are at least some contexts in which these relations hold. We sorely need studies that pit sentiment measures against each other and interrogate how context affects correspondence between sentiment, affect, and psychopathology (e.g., Massachi et al., 2020). These are crucial steps in developing a more robust science that can make concrete predictions about where, when, and for whom linguistic sentiment measures reflect affect and psychopathology.

Perhaps language can tell us not just whether people are feeling good or bad but also how they are managing or regulating their emotions. Indeed, there are deep connections between our words and our thoughts, as well as between our thoughts and our emotions (e.g., Beck’s (1991) cognitive model of the mind). There are many methods for regulating one’s emotions, but a key helpful strategy is cognitive reappraisal, in which one reinterprets a situation to alter its emotional impact (Gross, 1998). Interestingly, key lines of research on linguistic distancing conducted on English-speaking participants in Western settings show that one can enact this process merely by changing how one talks. Specifically, people feel better when they make their language more distant (i.e., swapping the word “I” for one’s name or switching verbs from present tense to past or future tense; Kross et al., 2014; Nook et al., 2017; Orvell et al., 2021). Distancing one’s language seems to induce psychological distancing (a specific reappraisal method; Kross & Ayduk, 2008), leading to less intense emotions, with shifts evident even at the neural level of analysis (Moser et al., 2017). But this relationship is also bidirectional: regulating emotions spontaneously distances language. Indeed, several controlled laboratory studies have now shown that you can predict how successfully someone has regulated their emotions merely by examining how much they distanced their language during regulation (Nook et al., 2017; Nook et al., 2020; Shahane & Denny, 2019). Perhaps most importantly, these findings seem to translate to real-world impact. Individual studies and meta-analyses of laboratory tasks (e.g., responses to images or a structured interview) and naturalistic in vivo exchanges (e.g., random samples of daily speech, Facebook posts, text messages) show that people who tend to distance their language more report lower internalizing symptoms (Berry-Blunt et al., 2021; Edwards & Holtzman, 2017; McNeilly et al., 2023; Stade et al., in press). Finally, youth and adults who distance their language more during psychotherapeutic interactions (in both individual therapy and single-session intervention settings) show better outcomes (Cohen et al., 2022; Nook et al., 2022).

Connecting these lines of work prompts the exciting idea that linguistic distancing can serve as a measure of emotion regulation and mental health, as well as a tool for down-regulating negative emotions. Although these studies benefit from strong alignment between theoretical grounding, laboratory experimentation, and clinical translation, open questions and key assumptions must be addressed if we are to conclude that distancing does indeed reflect processes central to psychopathology and thus a tool for wide-scale detection and intervention efforts. These include (i) uniting the lines of work above by demonstrating that linguistic distancing tracks lower internalizing symptoms because it reflects improved emotion regulation, (ii) ruling out potential third variables that could explain distancing-regulation and distancing-psychopathology associations, (iii) testing whether distancing one’s language causally impacts symptom reduction, (iv) investigating what aspect of friends’ or therapists’ language might contribute to linguistic distancing and/or treatment outcomes, (v) testing whether distancing-regulation relations exist across cultures or languages, (vi) identifying the biological or neural bases of these relationships, and (vii) developing strategies for integrating and disseminating this line of research to improve mental health at large scales. Addressing these broader questions represents a truly exciting opportunity to integrate other areas of affective science (e.g., research on interpersonal emotion regulation; Nook et al., in press; Sahi et al., 2021; Zaki & Williams, 2013) as well as other disciplines and subdisciplines (e.g., cultural psychology, linguistics, and intervention science).

Finally, people use language to parse their emotional experiences into categories. Although some people can identify subtle distinctions in their affective experiences (e.g., separating “annoyance” from “frustration”), others do not typically make these distinctions and may even struggle to see how different emotion words have different meanings (e.g., thinking of “sad” and “mad” as merely meaning “feeling bad”). This ability to specifically identify one’s feelings is referred to as emotion differentiation or emotion granularity (Barrett et al., 2001; Erbas et al., 2014; Kashdan et al., 2015; Thompson et al., 2021). Scientists measure emotion differentiation by asking people to rate how much they feel different emotions of the same valence. Differentiation scores are computed by reverse-scoring the intraclass correlation of these ratings: High correlations between these ratings indicate that people essentially use all of these emotion words identically, whereas low correlations indicate that they think of each as a unique emotional experience. Emotion differentiation scores particularly for negative emotions-have now been widely associated with healthy emotion regulation (Barrett et al., 2001; Kalokerinos et al., 2019), mental health in both youth and adults (Demiralp et al., 2012; Liu et al., 2020; Nook, 2021; Nook, Flournoy et al., 2021; Nook, Satpute et al., 2021; O’Toole et al., 2020; Seah & Coifman, 2022), and even treatment response (Lazarus & Fisher, 2021).

However, unlike the other constructs reviewed here, emotion differentiation does not currently have a purely linguistic measure. Because the construct is focused on how people use emotional words (i.e., do they understand “anger” and “sadness” as different?), studies currently require analyzing how participants use self-report emotion scales. Attempts at linguistic measures (i.e., scoring words as differentiated [“irate”] vs. undifferentiated [“bad”]) unfortunately do not converge with conventional measures (Ottenstein & Lischetzke, 2020; Williams & Uliaszek, 2022), and other studies show that using emotion words to label our emotions might make it harder to manage those emotions (Nook, Flournoy et al., 2021; Nook, Satpute et al., 2021; Vine et al., 2019; Vine et al., 2020). Thus, inventing a purely linguistic measure of this important ability remains a puzzle for affective scientists to solve. One idea would be to use a word embedding approach (which quantifies the “meaning” of a word based on the company it keeps; Firth, 1957) and test whether people with high emotion differentiation have emotion words with highly dissimilar meanings (i.e., the words they tend to produce around the word “anger” differ substantially from those they produce around “sadness”) compared to people with low emotion differentiation (who accompany “anger” and “sadness” with the same set of words; see Charlesworth et al., 2021 for a recent application of this method). Developing this linguistic measure offers an exciting opportunity to develop a useful linguistic tool and clarify basic questions about how language influences emotional experience.

Affective and clinical scientists have identified key linguistic constructs that show promise in measuring and/or shaping how people feel. These discoveries set the stage for tools that can detect psychopathology at large scales using language alone, and they suggest that changing one’s language could amplify healthy emotion regulation and reduce psychopathological symptoms. Nonetheless, open questions abound regarding the contextual generalizability, robustness, predictive utility, and underlying mechanisms for these phenomena. Future years should focus on further integrating computational techniques (e.g., machine learning; Franz et al., 2020), systematically identifying the contexts when these phenomena do and do not validly measure emotion, developing automated algorithms that can diagnostically predict emotion and symptom levels from language alone, incorporating dyadic and interpersonal processes, and testing the causal impact of shifting language on emotion and mental health. Additionally, the recent explosion of large language models (e.g., ChatGPT) opens countless new avenues for research, from automatically coding language samples (Rathje et al., 2023) to testing how people perceive affect from the computer- vs. human-generated language (Ayers et al., 2023). Careful integration of the points raised in this review will be important in further developing these tools. For example, it is crucial to clarify what exactly these models are coding from language (i.e., a person’s affective experience vs. the affect that is semantically associated with the language the person is using; Itkes & Kron, 2019).

Language is ubiquitous in our daily lives, and it comprises the key medium through which clinicians perform psychotherapy. As such, integrating across the specific phenomena discussed here and developing a clear understanding of the relationships between language, emotion, and health can advance affective science on both theoretical and applied fronts. Imagine a future where linguistic analyses could alert teachers to which students are experiencing bullying or academic anxiety, where changes in affective language could be a simple and widely-sampled outcome measure for determining which therapies are most effective for which patients, where therapists could receive immediate feedback about their patient’s wellbeing from their language alone, or where therapists in training could receive precise instructions for what exactly to say to have the most beneficial impact on their patients. There are certainly many challenges we must overcome to build a mature science that can achieve such visions, but nonetheless, the future is bright.

## Additional Information

## Data Availability

Not applicable.
